# Multiple Critical Periods for Rapamycin Treatment to Correct Structural Defects in *Tsc-1*-Suppressed Brain

**DOI:** 10.3389/fnmol.2018.00409

**Published:** 2018-11-08

**Authors:** Rebecca L. Cox, Froylan Calderon de Anda, Tomer Mangoubi, Akira Yoshii

**Affiliations:** ^1^Department of Brain and Cognitive Science, Massachusetts Institute of Technology, Cambridge, MA, United States; ^2^Feil Family Brain and Mind Research Institute, Weill Cornell Medical College, New York, NY, United States; ^3^Center for Molecular Neurobiology Hamburg, Research Group Neuronal Development, University Medical Center Hamburg-Eppendorf, Hamburg, Germany; ^4^Department of Anatomy & Cell Biology, University of Illinois at Chicago, Chicago, IL, United States

**Keywords:** tuberous sclerosis complex, neuronal migration, synapse formation, critical period, rapamycin

## Abstract

Tuberous sclerosis complex (TSC) is an autosomal dominant neurogenetic disorder affecting the brain and other vital organs. Neurological symptoms include epilepsy, intellectual disability, and autism. TSC is caused by a loss-of-function mutation in the *TSC1* or *TSC2* gene. These gene products form a protein complex and normally suppress mammalian target of rapamycin (mTOR) activity. mTOR inhibitors have been used to treat subependymal glioma (SEGA) that is a brain tumor characteristic of TSC. However, neuropathology of TSC also involves dysregulated cortical circuit formation including neuronal migration, axodendritic differentiation, and synapse formation. It is currently unknown to what extent mTOR signaling inhibitors correct an alteration in neuronal morphology that have already formed prior to the treatment. Here, we address the efficacy of rapamycin treatment on neuronal migration and dendrite formation. Using *in utero* electroporation, we suppressed *Tsc1* expression in a fraction of neuronal progenitor cells during the fetal period. In embryonic brain slices, we found that more *Tsc1*-suppressed cells remained within the periventricular zone, and rapamycin treatment facilitated neuronal migration. Postnatally, *Tsc1*-suppressed pyramidal neurons showed more complex branching of basal dendrites and a higher spine density at postnatal day (P) 28. Aberrant arborization was normalized by rapamycin administration every other day between P1 and P13 but not P15 and P27. In contrast, abnormal spine maturation improved by rapamycin treatment between P15 and P27 but not P1 and P13. Our results indicate that there are multiple critical windows for correcting different aspects of structural abnormalities in TSC, and the responses depend on the stage of neuronal circuit formation. These data warrant a search for an additional therapeutic target to treat neurological symptoms of TSC.

## Introduction

Tuberous sclerosis complex (TSC) is an autosomal dominant genetic disorder that involves multiple organs including brain, kidney, lung, and heart (Crino et al., 2006). Neurological symptoms of TSC include epilepsy, intellectual disabilities, and autistic behaviors. TSC is caused by loss-of-function mutations in either *TSC1* or *TSC2* (Kandt et al., 1992; European Chromosome 16 Tuberous Sclerosis Consortium, 1993; van Slegtenhorst et al., 1997). The TSC-1/TSC-2 protein complex (Plank et al., 1998; van Slegtenhorst et al., 1998) negatively regulates the mammalian target of rapamycin (mTOR) pathway, which is triggered by growth factors as well as nutrients and regulates protein synthesis, autophagy, transcription cell growth, cell proliferation, cell motility (Hay and Sonenberg, 2004; Sarbassov et al., 2005).

Neuropathological features of TSC include cortical tubers, subependymal nodules, glioradial fibers, subependymal giant astrocytoma (SEGA). Cortical tubers are hamartomatous tissues and are thought to be a migrational defect of neuronal progenitors (Crino, 2004; Marcotte and Crino, 2006). Neuronal migration has been studied in mouse models of TSC, such as two *Nestin*-promoter driven conditional *Tsc1* knockout mice targeting pyramidal cells, interneurons and glial cells. These models successfully recapitulated pathological features such as subependymal nodule-like lesion (Zhou et al., 2011) or cortical tuber giant cells (Goto et al., 2011).

A recent postmortem study in humans examined non-tuber cortical areas and identified “dyslamination” characterized by an altered radial orientation of pyramidal cells, blurring of laminar boundaries, and disruption of cortical columnar architecture, isolated balloon cells and heterotopic neurons inside subcortical white matter (Marcotte et al., 2012). Indeed, *Emx1-Cre* x *Tsc1*^*loxp*/*loxp*^ mice, which show *Tsc1*-deletion in forebrain pyramidal neurons starting from an early embryonic age, appear to lose cortical lamination without tubers or other obvious focal lesions (Magri et al., 2011; Carson et al., 2012). These findings suggest that TSC brains have diffuse and more subtle abnormalities outside of tubers than previously thought. Furthermore, there are also pathological findings that involve postmitotic neurons or precursor cells at the microscopic level. Specifically, animal models of TSC also showed abnormal axonal growth (Choi et al., 2008; Nie et al., 2010), and dendritic spine pruning (Tang et al., 2014). The mTOR pathway also plays critical roles in synaptic function (Hoeffer and Klann, 2010; Yoshii and Constantine-Paton, 2010). For example, *Tsc2* heterozygous mutant mice have impaired late long-term potentiation (L-LTP) and long-term memory (Ehninger et al., 2008). *Tsc1*-suppressed neurons have impaired α-amino-3-hydroxy-5-methyl-4-isoxazolepropionic acid receptor (AMPAR) currents (Tavazoie et al., 2005) and long-term depression (LTD) (Bateup et al., 2011). These microscopic structural changes and functional alterations underlie neurological disabilities in TSC.

Mutations in *TSC* genes result in overactivation of mTOR. Therefore, mTOR suppression by rapamycin or its derivatives corrects TSC pathophysiology and other mTOR-related disorders (Lipton and Sahin, 2014). For example, everolimus, a rapamycin derivative, reduced the size of SEGA and improved seizure control (Krueger et al., 2010; French et al., 2016). Neuronal circuit formation is a sequence of distinct developmental processes which include neurogenesis axonal growth, dendritogenesis, and synaptogenesis. In the rodent cortex, neurogenesis starts around E11 and ends around E17 (Takahashi et al., 1996; Caviness et al., 2009; Greig et al., 2013), and dendritic arborization occur in the first 2 weeks (Cline, 2001; Wong and Ghosh, 2002). Spine formation and pruning are maximal during the critical period, which starts P16 peaks at P28 and decline from P33 (Hensch, 2005). It is likely that the response to rapamycin is maximal while the abnormal morphology is formed. However, it remains unclear whether each of these cellular processes has a sensitive period to respond to the mTOR inhibitor treatment.

Here, we address the efficacy of rapamycin treatment on neuronal migration, the formation of dendrites and spines. Using *in utero* electroporation, we transferred a DNA construct encoding Cre recombinase tagged with green fluorescent protein (Cre-GFP) into E 15.5 neuronal progenitor cells in a *Tsc1*^*fl*/*fl*^ mouse fetal brain and suppressed the gene expression in a group of cells that are born around the same time. In embryonic brain slices, we found that more *Tsc1*-suppressed cells remained within the periventricular zone and that rapamycin treatment facilitated neuronal migration. Postnatally, the lamination pattern of *Tsc1*-suppressed neurons was widened and scattered more than WT cells. Further, *Tsc1*-suppressed pyramidal neurons showed more complex branching of basal dendrites, which was normalized by rapamycin administrations between postnatal day (P) 1 and P13 but not between P15 and P27. In contrast, abnormal spine maturation improved with rapamycin between P15 and P27 but not between P1 and P13. These results suggest that there is a critical time window during neuronal circuit formation to correct abnormal neuronal morphology in TSC.

## Materials and methods

### Animal

This study was carried out in accordance with the principles of the Basel Declaration and recommendations of MIT, UIC, and NIH guidelines on the humane care of animals. The protocol was approved by the MIT- and UIC-IACUC. All animal manipulations were approved by the MIT- and UIC-IACUC and were performed in accord with its guidelines. *Tsc1*^*loxp*/*loxp*^ mice (Jackson Laboratory, #005680) were kept under 12 h light/dark cycle. Rapamycin was injected every other day (6 mg/kg/dose) (Meikle et al., 2008).

### *In utero* electroporation

Timed pregnant mothers were anesthetized with 2–3% isoflurane and oxygen. Following laparotomy, the uterus was externalized and the lateral ventricle of E15.5 embryos was injected with 1–2 μg Cre-GFP alone or in combination with 0.1–0.2 μg DiO-YFP. Using an ECM 830 apparatus (Harvard Apparatus, Holliston MA), brains were electroporated with five 30 V, 50-ms pulses at intervals of 950 ms. After recovery, pregnancies continued, and pups were delivered normally.

### Organotypic slice cultures

Mouse embryos were electroporated at embryonic day 15 (E15), and acute coronal brain slices (240 μm) were prepared at E17 and E18. Occipital slices were transferred onto slice culture inserts (Millicell) in cell culture dishes (35 × 10 mm; Corning) with Neurobasal medium (Invitrogen) containing the following: B27 (1%), glutamine (1%), penicillin/streptomycin (1%), horse serum (5%), and N2 (1%). Slices were used for imaging (1–2 h after slicing) or for pharmacological treatments (incubated at 37°C in 5% CO_2_, for 1 day). A subset of slices was incubated with the medium containing rapamycin (100 μM).

### Time-lapse imaging

Cre-GFP- and mCherry-positive cells were imaged on an inverted Nikon microscope (TE 2000-S) with a 20 × objective lens [numerical aperture (NA) 0.45]. mCherry was added to ensure fluorescent signal detection during serial imaging. During the time-lapse imaging, slices were kept in an acrylic chamber at 37°C in 5% CO_2_. We captured time-lapse images with a Cool SNAP EZ camera (Roper Scientific) using NIS-Elements software (Nikon).

## Analysis of neuronal migration velocity

The neuronal migration velocity was measured using a plugin for ImageJ (Mouse Tracker, programmed by P. Malatesta, IST Genova) that allows tracking the cell position over time. Using the coordinates obtained with ImageJ, the velocity was calculated with Excel (Microsoft) (de Anda et al., 2010).

### Immunohistochemistry and confocal microscopy

**Figure 1:**
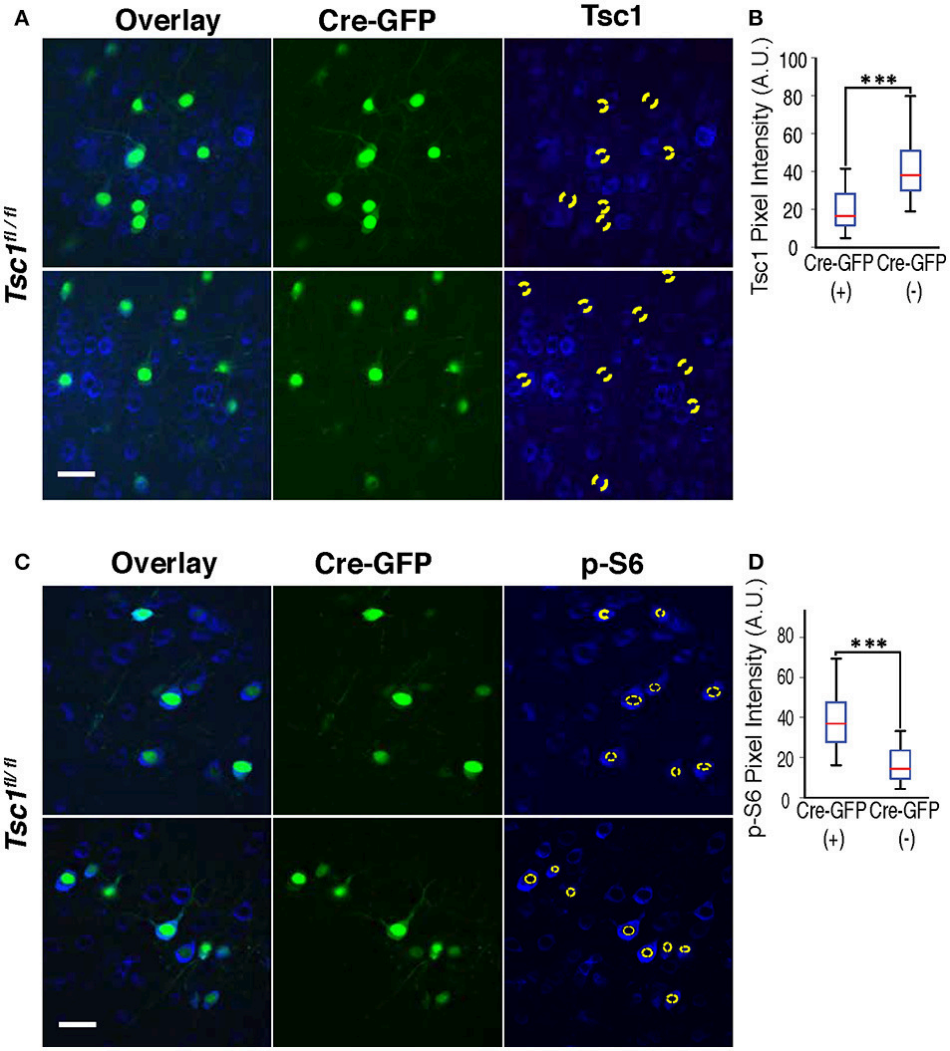
Immunohistochemistry of Tsc1 and p-S6. **(A,B)** In *Tsc1*^*fl*/*fl*^ mouse brains, neurons expressing Cre-GFP (indicated with a dotted circle) shows suppression of Tsc1 protein as compared with neurons without Cre-GFP. **(C,D)** Immunohistochemistry of p-S6 protein shows increases in neurons expressing Cre-GFP (labeled with a dotted circle), indicating overactivation of mTOR signaling. A magnification of the neuron encircled by a white box is shown at the bottom and depicts the cytosol (a space between two yellow dashed lines), which is defined as ROI to measure immunolabels. In **(A,C)**, scale bars indicate 50 μm. In **(B,D)**, ^***^P < 0.001, and sidebars show the range of data set.

Following transcardiac perfusion with 4% paraformaldehyde in phosphate buffered saline (pH7.4), brains are post-fixed, trimmed, embedded in 2% low temperature-melt agarose with PBS and 7% sucrose, and sectioned on a vibratome in the coronal plane at 50 μm for immunohistochemistry and100 μm for Sholl and spine analyses. A cryostat was used for thinner sections of fetal brains. Sections are permeabilized in PBS/4% donkey serum/1% Triton X100 at room temperature 10 min. After rinsing in PBS for 15 min three times, sections are reacted overnight with primary antibodies in PBS/4% donkey serum/0.5%Ttriton X100 at room temp. The following primary antibodies were used; TSC1 (Cell Signaling Technology, #4906); phosphorylated S6 (Cell Signaling Technology, #4858); and Brn2 (Cell Signaling Technology, #12137). After rinsing with PBS (15 min, three times), sections were incubated in an Alexa 568-conjugated secondary antibody overnight at room temp and finally rinsed in PBS (15 min, three times). Images were captured using a 40 x objective lens under identical settings with a Nikon PCM 2000 confocal microscope. The visual cortex was identified on coronal sections using the Paxinos atlas. The *Z*-series of optical sections taken at intervals of 0.5 μm were reconstructed using the same setting. ImageJ was used to measure pixel intensity of the immunolabels in the cytosol. The regions of interest (ROIs) were selected using freehand line tool, then signals in neclei were subtracted. An example of the cytosol is selected with a white box in Figure 1C and magnified at the bottom. Averaged pixel intensity was calculated by dividing total pixel intensities with the pixel area of cytosol. Similarly, averaged intensity of Cre-GFP signal was measured. A cell was considered Cre-GFP positive when its averaged Cre-GFP intensity in the nucleus was above 100. Immunolabels of TSC1 and phosphorylated S6 were compared between neurons with and without Cre-GFP.

To measure mTOR activity, immunostaining was performed using an antibody to phosphorylated serine 240/244 of the S6 ribosomal pro- tein (pS6). Serial Z-stacks were acquired under the same settings for the ipsilateral and contralateral hemispheres of coronal sections. Because layer 2/3 cortical neurons project to the contralateral hemisphere, mRFP^+^ axons were followed to measure pS6 levels in cells of the non-electroporated region. ROIs were generated using an elliptical selection tool, and average intensi- ties for each ROI were determined. The ROI did not include the nucleus that had low pS6 staining. For both cell size and pS6 staining measurement, 3 sections per mouse and 38–111 cells per section were analyzed.

### Morphological analysis

Neurons with a strong signal whose cell body was entirely contained within the slice were selected for morphological analyses in the brains of P28 animals. Z-stacks were stitched together in the XY plane such that the full span of the neurons projections were included in the composite image. Neurolucida 8 software (MBF Bioscience) was used for quantitative analyses of neuronal morphology, including Sholl analysis of apical and basal dendrites, and largest cell body area (Yoshii et al., 2011). Neurons were blindly traced and analyzed based on spherical shells concentric with the somal centroid and spaced at a 5μm interval. The number of intersections and nodes were counted from the soma to the distal end of dendritic branches. For spine density, basal dendritic branches within the interval between 15–40 μm and number of spines were counted to calculate the spine density (number of spines per μm).

### Statistics

**Figure 2:**
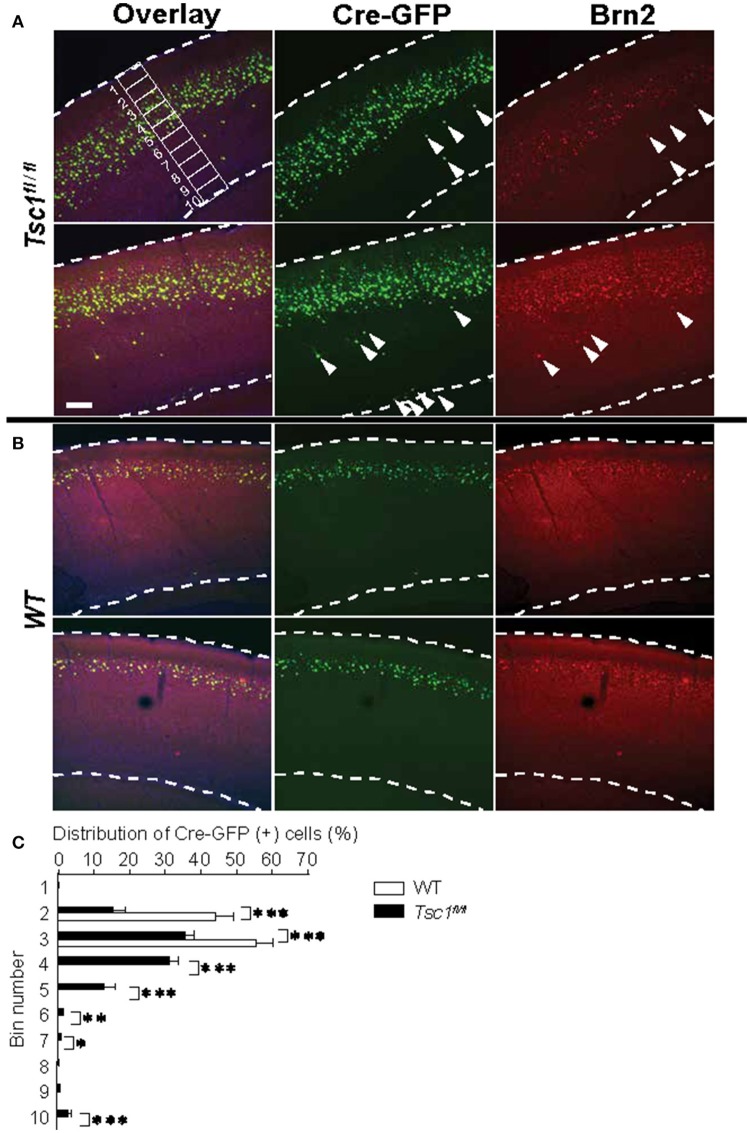
*Tsc1*-suppressed neurons show a scattered distribution and some cells are mislocalized outside of layer 2/3. **(A)** In *Tsc1*^*fl*/*fl*^ mouse brains, the distribution of Cre-GFP (+) neurons is scattered. Arrowheads indicate mislocalized cells and some of them are positive for the layer 2/3 marker Brn2. An example of 10 segments is shown. **(B)** In WT brains, the distribution of neurons that were electroporated with Cre-GFP at E15.5 is consistent with layer 2/3. **(C)** The graph shows averaged percentage of Cre-GFP (+) cell in 10 segments as shown in **(A)**. Cre-GFP (+) neurons in *Tsc1*^*fl*/*fl*^ mouse brains is more widely distributed than those in WT. ^*^P < 0.05.^**^P < 0.01.^***^P < 0.001. In **(A,B)**, dashed lines indicate the superficial and ventricular surface of the visual cortex. Scale bar, 50 μm.

**Figure 3:**
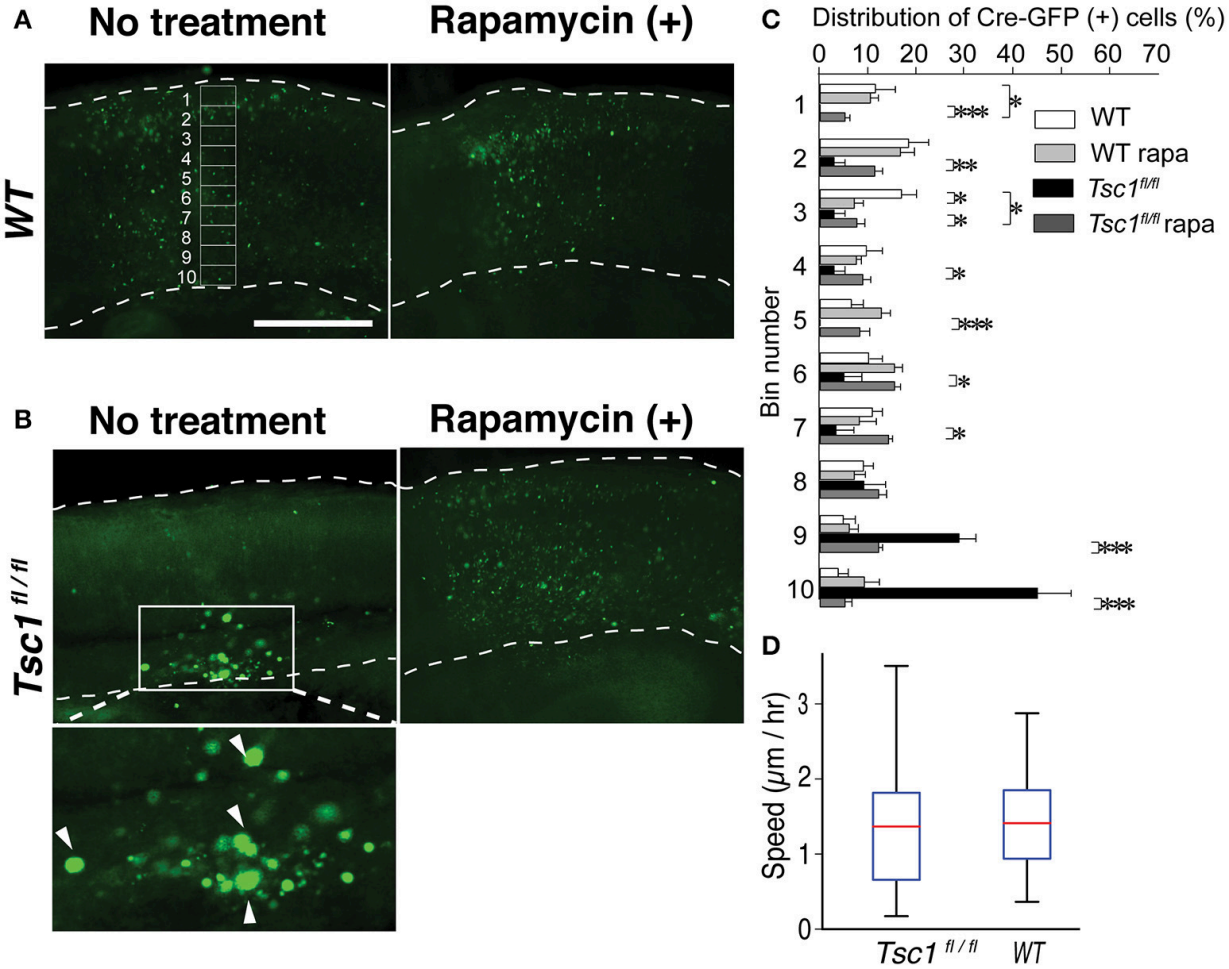
Rapamycin treatment of the fetal brain slice corrected migration of *Tsc1*-suppressed neuronal progenitors. **(A)** Fetal cortical slices that were prepared at E18.5. An example of 10 segments is shown. Scale bar, 50 μm. **(B)** In *Tsc1*^*fl*/*fl*^ slice, Cre-GFP (+) cells without rapamycin treatment appear to be balloon cells (arrowheads in the magnified image) and stay within the ventricular zone. **(C)** In WT slice with or without rapamycin treatment, rapamycin treatment resulted in subtle distribution changes. In *Tsc1*^*fl*/*fl*^ slice treated with rapamycin (100 μM), Cre-GFP (+) cells migrate to the cortical plate and are similar to progenitor cells in WT (except for Bin 1 and 3). Approximately 70% of *Tsc1*-suppressed cells without rapamycin treatment is localized in Bin 9 and 10. **(D)** The velocity of migrating cells is comparable between *Tsc1*^*fl*/*fl*^ and WT brains. In **(C)**, ^*^P < 0.05. ^**^P < 0.01. ^***^P < 0.001.

**Figure 4:**
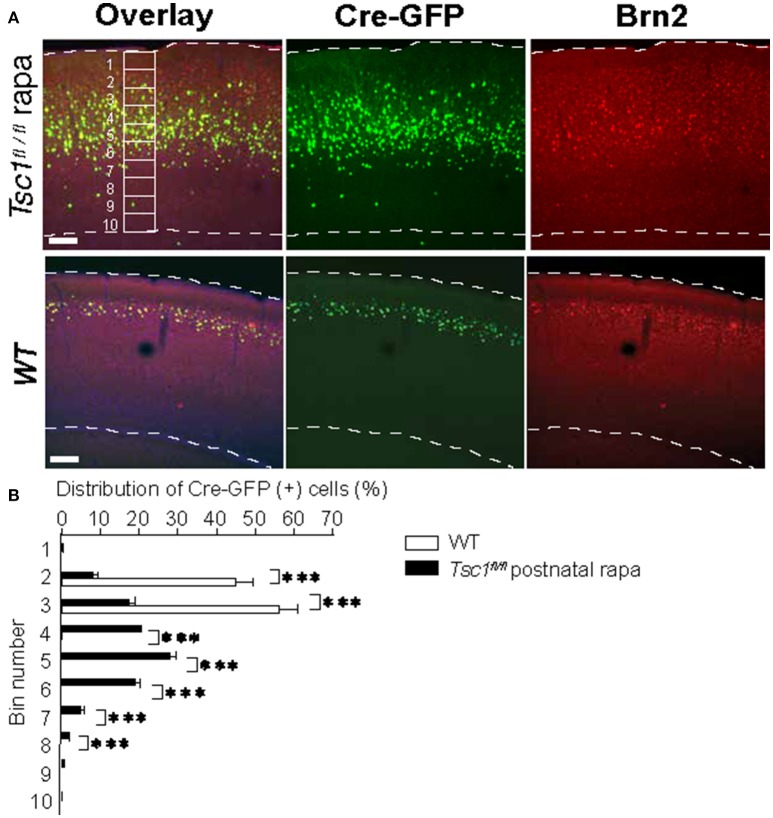
Postnatal administration of rapamycin does not correct the aberrant migration pattern of *Tsc1*-suppressed neurons. **(A)** Representative images of the visual cortices collected from a *Tsc1*^*fl*/*fl*^ mouse with postnatal rapamycin treatment and a WT animal. An example of 10 segments is shown. **(B)** In *Tsc1*^*fl*/*fl*^ animals that treated with rapamycin postnatally, the distribution of neurons electroporated with Cre-GFP at 15.5 is wider than that in WT cortex. ^*^P < 0.05. ^**^P < 0.01. ^***^P < 0.001.

**Figure 5:**
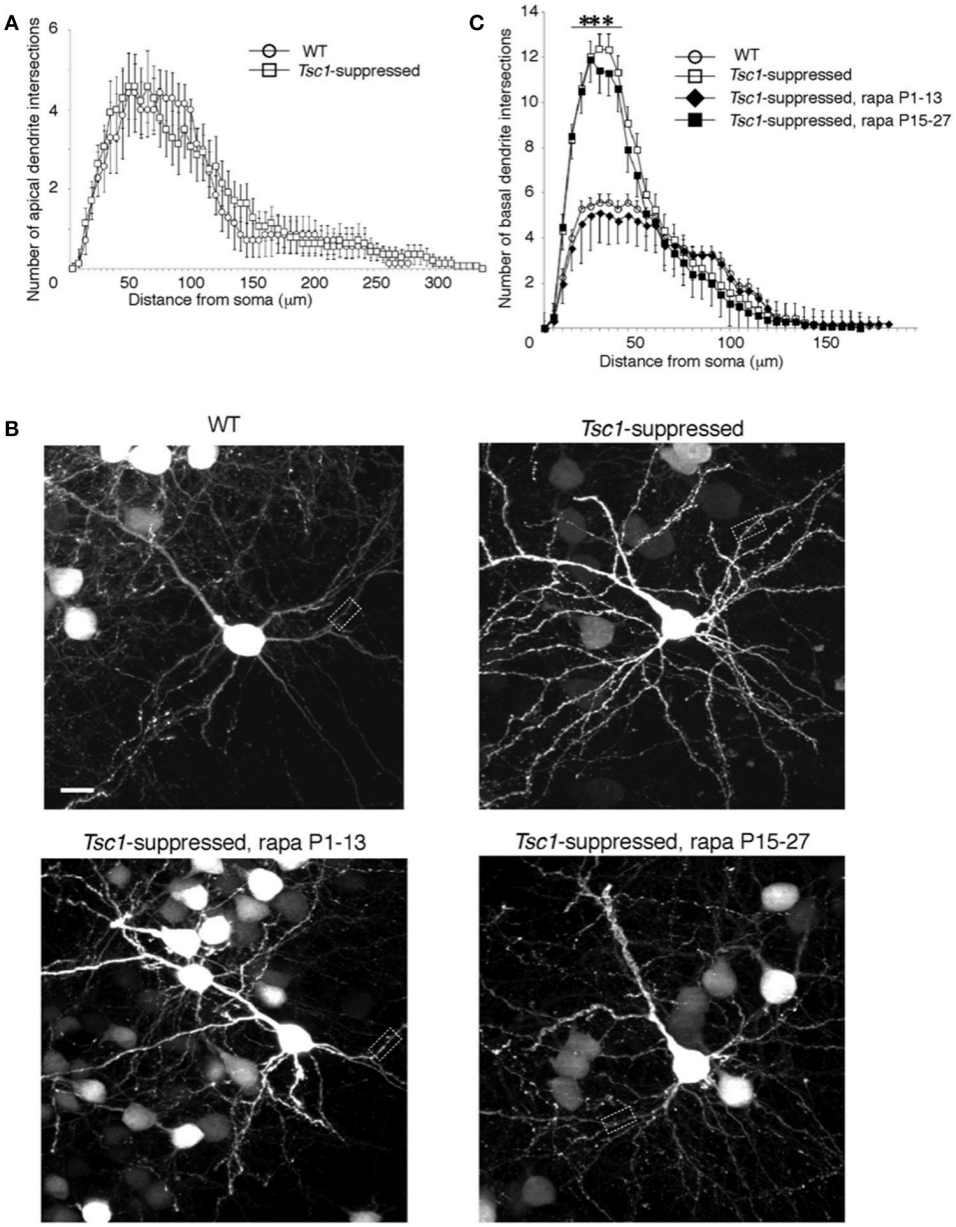
Sholl analysis of electroporated pyramidal neurons. **(A)** There was no significant difference between *Tsc1*-suppressed and WT neurons in numbers of intersections throughout the apical dendrite. **(B)** WT and *Tsc1*-suppressed neurons with and without rapamycin treatment. Dashed boxes are magnified in Figure 6A. Scale bar, 10 μm. **(C)** Basal dendrites of *Tsc1*-suppressed neuron had higher intersection numbers than WT cells between 15 and 40 μm from the soma. In *Tsc1*-suppressed neurons, rapamycin treatment between P1 and P13 but not between P 15 and 27 reduced the basal dendrite arborization. ^***^P < 0.001 Error bars represent s.e.m.

**Figure 6:**
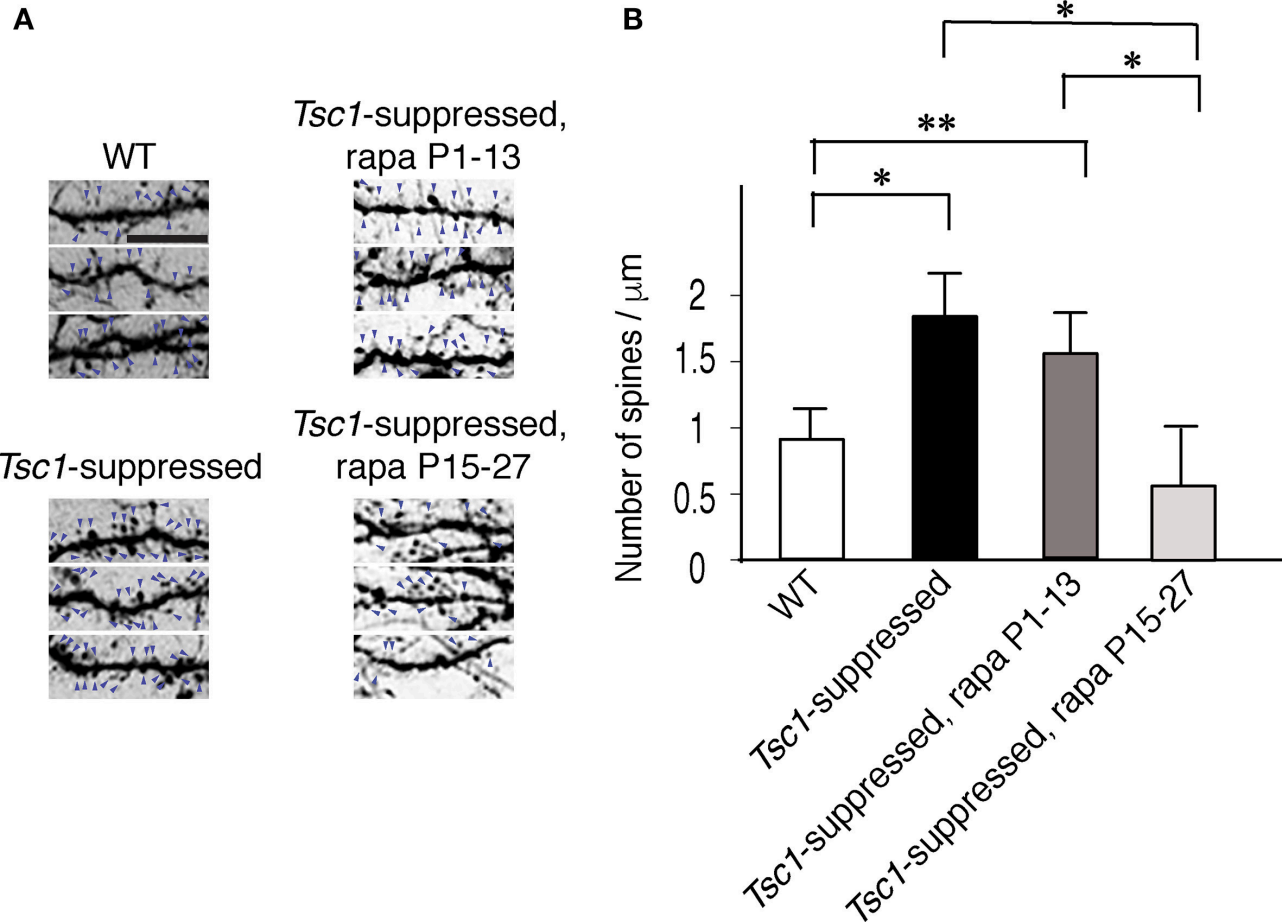
Spine density of basal dendrites in electroporated pyramidal neurons. **(A)** Basal dendritic segments that are encircled with dashed boxes in Figure 5B. The signal density of each image is inverted for an improved visualization. Arrows indicate spines. Scale bar, 5 μm. **(B)** *Tsc1*-suppressed neurons have a higher spine density than WT cells at P28. Rapamycin treatment between P15 and P27 reduced spine density in *Tsc1*-suppressed neurons while neurons treated between P1 and P13 did not show a change in spine density. ^*^P < 0.05;^**^P < 0.01.

For immunohistochemical analysis, Wilcoxon Rank sum test was used. For neuronal migration and neuronal morphological analyses, a Student's *t*-test was used for comparison of two groups. One-way ANOVA with *post-hoc* Tukey tests was used for comparisons of more than two groups. *P* < 0.05 were considered significant and indicated as ^*^ in graphs. *P* < 0.01 and *p* < 0.001 are indicated as ^**^ and ^***^. Numeric data are presented as average ± s.d. in the Results section. Error bars in Figures 1–**6** represent s.e.m. In other figures, sidebars represent the range of data set.

## Results

To examine the migration of *Tsc1*-suppressed neuronal progenitor cells, we combined a Cre-lox recombination system and *in utero* electroporation. Specifically, we used a *Tsc1*^*fl*/*fl*^ mouse which has exon 17 and 18 flanked with Lox sequences and electroporated a DNA construct encoding Cre-GFP into wild-type (WT) or mutant fetuses at embryonic day 15.5 (E 15.5).

We performed Immunohistochemistry in both WT and mutant brains at P28 and confirmed that Cre-GFP expressing neurons in the mutant showed suppression of the TSC1 protein (Figures 1A,B) and increased phosphorylation of ribosomal protein S6 (p-S6) (Figures 1C,D), indicating an enhanced mTOR signaling as a result of suppressed TSC1 function.

### Cortical lamination is disorganized in TSC

Next, we examined the distribution of Cre-GFP positive neurons. In WT, Cre-GFP positive neurons were localized in cortical layer 2/3 when the DNA construct was electroporated in E 15.5. Remarkably, mutant brains showed a scattered distribution of Cre-GFP positive neurons (Figure 2A) as compared to normal layer 2/3 distribution in WT (Figure 2B). A minority of cells remained in deeper layers or the junction of layer 6 and the white matter (see arrowheads in Figure 2A) as previously reported (Feliciano et al., 2011). We further analyzed depth ratio by dividing the distance of each cell from the cortical surface with the cortical thickness. *Tsc-1-*suppressed cells showed significantly deeper distribution than WT (Figure 2C: *p* < 0.001, *N* = 300 cells each from three WT and five *Tsc1*^*fl*/*fl*^ animals). Some *Tsc-1-* suppressed cells were localized in a deeper layer even though they express Brn2, a marker protein for layer 2/3 cortical neurons (Figure 2A, see cells indicated by arrows in the *Tsc1*^*fl*/*fl*^ panel). This observation is consistent with disorganized cortical layer formation that is observed in other *Tsc-1* knockout mouse models and postmortem brains of patients with TSC (Magri et al., 2011; Carson et al., 2012).

To further study the neuronal migration defect in the cortex of TSC, we performed live cell imaging of fetal cortical slices. We electroporated a DNA construct encoding Cre-GFP into the *Tsc1*^*fl*/*fl*^ fetal cortices at embryonic day 15.5 (E 15.5). We made cortical slices at E17.5 and incubated them with medium with or without 100 μM rapamycin for 24 h. Then, we fixed the slices and imaged them using a confocal microscope (Figures 3A,B). We divided the cortical plate into 10 segments and quantified the distribution of migrating neurons (Figure 3C). In WT slices, Cre-GFP positive neurons migrated to the cortical plate at E18.5 *in vitro* (Figures 3A,C). Rapamycin treatment did not substantially affect migration of WT neurons. In contrast, ~70% (see Bin 9 and 10 in Figure 3C) of *Tsc1-*suppressed neurons were still localized in the ventricular or intermediate zones at E18.5 (Figures 3B,C). Importantly, the mispositioning of neurons at E18.5 can be improved by rapamycin treatment although fewer *Tsc1-*suppressed cells migrated to the superficial region of the cortical plate than WT neurons (see Bin 1 and 3 in Figure 3C). We also performed live imaging of slices prepared from E17.5 to measure migration velocities. While the majority of *Tsc1-*suppressed neurons remained in the ventricular or intermediate zones, ~20–30% of *Tsc1-*suppressed neurons were migrating. We measured velocity of the migrating neurons and found that averaged speed was comparable between WT and *Tsc1*-suppressed cells (Figure 3D, Videos 1, 2). These results indicate that *Tsc1*-suppressed post-mitotic neurons remain in the ventricular zone/intermediate zone longer than WT neurons and that rapamycin normalizes the departure timing. However, once neurons leave the ventricular zone/intermediate zone, they migrate properly.

Previous studies show that prenatal rapamycin treatment improves the migrational defect in *Tsc-1* and *-2* conditional knockout mouse models (Anderl et al., 2011; Way et al., 2012). Consequently, we asked whether postnatal administration of rapamycin corrects disrupted mispositioning of layer 2/3 neurons. We injected rapamycin intraperitoneally every other day (6 mg /kg/dose) starting from P1 till P 27 and examined at P28. However, this treatment did not correct mispositioning of *Tsc1*- suppressed neurons, which remained broadly distributed (Figure 4). Collectively, these results suggest that the optimal timing to treat the dyslamination defect in the TSC1 cortex is prenatal when the majority of newborn neurons are migrating toward the cortical plate.

### Neuronal morphology of postnatal pyramidal neurons

We examined the neuronal morphology of WT and *Tsc1*- suppressed neurons in the occipital cortex at P28. In agreement with previous studies, *Tsc1*-suppressed neurons showed larger soma size (389.74 ± 70.84 μm^2^ in *Tsc1*^*f*/*fl*^ and 194.91 ± 45.6 μm^2^ in WT; *p* < 0.01 *n* = 9 from three brains in each genotype). Using Sholl Analysis, we examined both apical and basal dendrite of layer 2/3 pyramidal neurons at P28 (10 neurons from three brains in each condition). In apical dendrites, there were no significant differences in both numbers of intersections and nodes between the two genotypes except for one node at 100 μm, where the average number of intersections measured in the 100 μm shell is significantly different between the two conditions (Figure 5A). We were unable to analyze distal segments of apical (tuft) dendrites, which were often cut off in 100 μm sections. In comparison, the Sholl analysis of basal dendritic branches indicates that there were more intersections in the dendritic segments of *Tsc1*^*fl*/*fl*^ neurons than in WT neurons between 15 to 40 μm from the somal centroid (Figures 5B,C and Table 1).

Next, we asked whether rapamycin corrects aberrant dendritic morphology of layer 2/3 pyramidal neurons. We administered rapamycin intraperitoneally every other day (6 mg /kg/dose) in two different duration: P1 to P13 and P15 to P27. Exuberant branching of proximal basal dendrites was normalized by rapamycin treatment between P1 to P13 but not between P15 and P27 (Figure 5C). Finally, we also measured spine density of proximal basal dendrites (10 cells from three brains in each group), which is defined as the number of dendritic spines per μm, and found that it was significantly higher in *Tsc1*-suppressed neurons than WT (Figure 6A and Table 1). The increase in spine density was normalized by rapamycin treatment between P15 to P27 but not between P1 and P13 (Figures 6A,B).

**Table 1:** Summary of ANOVA analyses.

	**Source**	**Degree of freedom**	**Sum of square**	**Mean square**	**F**	***P*-value**
Figure 5						
15μm	Between groups	3	218.075	72.692	97.2825	0.0006
	Within groups	36	26.9	0.747	
	Total	39	244.975		
20μm	Between groups	3	380.675	126.892	122.4692	0.0009
	Within groups	36	37.3	1.036	
	Total	39	417.975		
25μm	Between groups	3	453.9	151.3	172.3671	0.0015
	Within groups	36	31.6	0.878	
	Total	39	485.5		
30μm	Between groups	3	486.5	162.167	278	0.0031
	Within groups	36	21	0.583	
	Total	39	507.5		
35μm	Between groups	3	467.275	155.758	247.0176	0.0026
	Within groups	36	22.7	0.631	
	Total	39	489.975		
40μm	Between groups	3	413.3	137.767	124.6131	0.0009
	Within groups	36	39.8	1.106	
	Total	39	453.1		
FIGURE 6						
	Between groups	3	9.139	3.046	21.9933	0.0001
	Within groups	36	4.986	0.139	
	Total	39	14.125		

These results indicate that the rapamycin effect to normalize abnormal dendritic morphology depends on the timing of developmental maturation. Specifically, exuberant dendritic branching responds to rapamycin treatment within the first two postnatal weeks when dendritic arborization occurs, and aberrant dendritic spine maturation is treatable between P15-27 when spine formation and pruning peaks during the critical period in the visual cortex (Hensch, 2005; Stryker and Stryker, 2012).

## Discussion

Neurological symptoms of TSC include epilepsy, developmental delays, and autistic behavior. Classic neuropathological features are cortical tubers with giant cells, subependymal nodules, subependymal giant cell astrocytoma, and white matter radial migration lines (Crino et al., 2006). Recent studies also show structural abnormalities and functional deficits at cellular and synaptic levels (Tavazoie et al., 2005; Meikle et al., 2007; Choi et al., 2008; Nie et al., 2010; Goto et al., 2011; Magri et al., 2011; Zhou et al., 2011; Carson et al., 2012; Tang et al., 2014). In the present study, we have systematically analyzed neuronal morphology in TSC and assessed rapamycin effect on neurogenesis, dendritogenesis, and spinogenesis. Using *in utero* electroporation, we suppressed *Tsc-1* expression in a fraction of neuronal progenitor cells. In a mouse brain, neurogenesis starts around E11 and ends around E17 (Takahashi et al., 1996; Caviness et al., 2009; Greig et al., 2013). WT neuroblasts born at E15.5 are properly located in cortical layer 2/3. However, *Tsc-1*-suppressed progenitor cells make a delayed departure from ventricular zone and become scattered postnatally. These *Tsc-1*-suppressed cells also express a marker protein for layer 2/3 despite their malpositioning to a deeper layer. Postnatally, *Tsc1*-suppressed neurons have more complex dendritic arborization and a higher spine density than WT. Importantly, each of these developmental abnormalities that are caused by enhanced mTOR pathway has a specific window of opportunity to respond to rapamycin. Namely, dyslamination must be corrected during neurogenesis, and postnatal rapamycin treatment will not correct the cortical malformation. Similarly, exuberant branching of basal dendrites is rectifiable only during the first 2 weeks postnatally while an increase in spine density responds to rapamycin treatment thereafter. These results suggest that there are multiple critical periods to correct morphological defects in TSC during neuronal circuit formation. Notably, *Tsc2-hGFAP* mouse also exhibits time-sensitive responses to rapamycin: *in utreo* treatment corrects abnormal neuronal migration that results from radial glia dysfunction and postnatal rapamycin administration is necessary to rescue myeliniation defects (Way et al., 2012).

### Migration defect in TSC

Cortical tubers are demarcated hamartomatous tissues that contain dysplastic abnormal and large neurons, including balloon cells. Tubers are thought to be a product of migrational defect. Perhaps the most significant and disabling feature of patients with TSC is chronic and progressive seizures. There is an ongoing controversy concerning how the number or size of cortical tubers (often referred to as “tuber burden”) is linked to the severity of neurological disabilities of TSC. There is evidence that a higher cortical tuber count is associated with lower intelligence and increased incidence of infantile spasms (Doherty et al., 2005). Also, EEG discharges highly correlate with tuber locations in magnetic resonance imaging (MRI), and surgical resection of tubers often reduces seizure episodes (Koh et al., 2000). Another study showed an altered expression pattern of glutamate receptors in human cortical tubers (Talos et al., 2008).

While some of the tubers may be epileptogenic, it is not known if this is true of all cortical tubers or dependent on the tuber size, location or disrupted morphology. In fact, other studies have observed no solid correlation between tuber burden and the degree or kind of the neurological phenotype: severity of seizures, cognitive disability, or autism (Wong and Khong, 2006). One issue that complicates the clinical view of TSC is that differences may exist depending upon components of the tuber burden (e.g., not only number but also size, location, or morphology of the tubers) (Marcotte et al., 2012) and the severity or type of neurological phenotype. Furthermore, several disabilities may coexist and influence with each other. For example, frequent seizures can exacerbate cognitive and behavioral functions. Consequently, TSC can present with a broad spectrum of symptoms despite apparently comparable “tuber burdens.” The biological bases for this range remain controversial. In fact, there is increasing evidence that non-tuberous TSC brain regions can also have dysregulated synaptic functions and play a critical role in the generation of abnormal electrical activity and epilepsy. For example, neuroimaging data indicate that cortical excitability can originate in regions near but not within cortical tubers in some TSC patients (Asano et al., 2000, 2004). Further, a recent postmortem study in humans examined non-tuber cortical areas and identified “dyslamination” characterized by an altered radial orientation of pyramidal cells, blurring of laminar boundaries, and disruption of cortical columnar architecture, isolated balloon cells and heterotopic neurons inside subcortical white matter (Marcotte et al., 2012). Taken, together, cortical tubers alone may not be sufficient to explain neurological symptoms, and microscopic abnormalities outside a tuber result in impaired circuit formation.

Two *Nestin*-promoter driven conditional *Tsc1* knockout mice targeting pyramidal cells, interneurons and glial cells successfully recapitulated pathological features such as subependymal nodule-like lesion (Zhou et al., 2011) or cortical tuber giant cells (Goto et al., 2011). On the other hand, *Emx1-Cre* x *Tsc1*^*loxp*/*loxp*^ mice, which show *Tsc1*-deletion in forebrain pyramidal neurons starting from an early embryonic age, appear to lose cortical lamination without tubers or other obvious focal lesions (Magri et al., 2011; Carson et al., 2012).

Using *in utero* electroporation and live cell imaging, we find a scattered distribution of *Tsc1*-suppressed neurons. Dyslamination results from the delayed departure of the mutant progenitors from the ventricular zone and is correctable with rapamycin treatment during neurogenesis. However, postnatal administration of rapamycin did not correct malpositioning of *Tsc1*-suppressed neurons. Our results indicate that mTOR inhibitor can correct the cortical lamination defect if it is given during corticogenesis. While the result suggests cortical tubers and dyslamination are potentially treatable with a mTOR inhibitor, the treatment is currently not feasible in a human embryo due to a concern for teratogenicity.

### Dendritic branching in TSC

mTOR signaling pathway plays a critical role in dendritogenesis. Inhibition of phosphatidylinositol-4,5-bisphosphate 3-kinase (PI3K)-Akt pathway, which is upstream of mTOR, reduces dendritic arborization (Jaworski, 2005). In contrast, phosphatase and tensin homolog (PTEN) is a negative regulator of the PI3K-Akt-mTOR pathway by increasing phosphatidyl 3- inositol. Thus, loss-of-function PTEN mutations cause upregulation of mTOR and are associated with autism, macrocephaly, and epilepsy (Butler et al., 2005; Herman et al., 2007; Hoeffer and Klann, 2010). Hippocampal CA1 pyramidal neurons of the *Pten* KO mouse exhibits exuberant arborization (Kwon et al., 2006). *Pten*-deleted pyramidal neurons in the layer 2/3 visual cortex of adult mouse showed an extension of apical dendrite length (Chow et al., 2009). We have limited Sholl analysis to proximal segments of apical dendrites since distal segments were often cut off in the 100 μm sections. However, the difference of apical vs. basal dendrites may also reflect the distinct roles between PTEN and TSC1 proteins. Aberrant dendritic branching has also been reported in *Tsc-1* and -*2* RNA interference model in hippocampal neurons although they show tortuous but not exuberant dendritic arbors (Tavazoie et al., 2005). Neurons in different brain regions and ages have different local connectivity, which is also likely to contribute to the interpretation. Nevertheless, abnormally elaborate dendritic arborization occur in response to up-regulation of mTOR signaling pathway.

Recent evidence suggests that each dendritic arbor process distinct information. For example, in a pyramidal neuron of the mouse visual cortex, each dendritic branch is tuned to a different orientation, and they are summated in the soma (Hausser, 2000; Jia et al., 2010; Grienberger et al., 2015). Further study is needed to address whether dendritic integration and sensory processing are dysregulated in *Tsc1*-suppressed neurons.

In mice, the final morphology of the dendritic tree is formed in the first 2 weeks of postnatal development during a period of maximum afferent innervation and synapse formation (Cline, 2001; Wong and Ghosh, 2002), then the large-scale dendritic structures become markedly stable (Trachtenberg et al., 2002; Holtmaat et al., 2006; Lee et al., 2006). We observe that rapamycin treatment is effective in correcting exuberant branching of proximal basal dendrites when it is administered between P1 to P13 but not between P15 and P27 (Figure 5). Our results indicate that there is a critical period for rapamycin response to normalize exuberant dendritic branching of *Tsc-1*-suppressed neurons that correspond to the first 2 weeks of postnatal life. Collectively, mTOR pathway is responsible for dendritic arborization in an early postnatal period before spine maturation occurs.

### Dendritic spine defect in TSC

In the rodent central visual system, experience-dependent synapse formation starts after eye-opening at P13, the onset of patterned vision (Yoshii et al., 2003, 2011; Yoshii and Constantine-Paton, 2007). Spine formation and pruning are maximal during the critical period, which starts P16 peaks at P28 and decline from P33 (Hensch, 2005). We find that *Tsc-1* suppressed neurons in the visual cortex have an increase in dendric spine density. Our observation is in line with a previous study showing pruning defect in TSC (Tang et al., 2014). We also find that the rapamycin effect on aberrant spine formation is optimal between P15 and P27 when activity-dependent synapse formation is at its peak.

Important questions that need to be addressed in the future are whether disorganized lamination and neuronal morphology also disrupt local and long-range connections, and whether mTOR inhibitor treatment can correct them. The balance between excitation and inhibition undergoes complex regulation within the local cortical circuitry. A recent study in WT mouse somatosensory cortex using optogenetics and electrophysiology showed that horizontal projections originating from layer 2/3 pyramidal cells suppress activities of adjacent cortical regions within the same layer by lateral inhibition while facilitating layer 5 neuron activity (Adesnik and Scanziani, 2010). It is likely that an excitatory-inhibitory balance in the horizontal and vertical circuits is altered in TSC. Another critical question is whether the dyslaminated cortex can still establish normal long-range connectivity (Normand and Rasband, 2015).

## Conclusion

Our study suggests that *Tsc-1* suppressed cortical neurons show alterations in cellular organization and differentiation and that each process has a distinct critical period in which rapamycin corrects the abnormal cellular process. Clinical studies have documented encouraging observations that rapamycin derivative such as everolimus is effective for not only controlling SEGA growth but also improves the overall outcome of seizure frequency (Krueger et al., 2010; French et al., 2016). Ideally, mTOR inhibitor should be started as soon as the diagnosis is made. However, even if the treatment is initiated shortly after birth, migration and dendritogenesis defects that have already occurred may be irreversible, and they may lead to a secondary effect during circuit formation. For example, proper lamination is essential for sensory processing (Adesnik et al., 2012). While rapamycin treatment is effective in correcting dendritic spine formation, our results suggest that there may be a limit in compensating for the structural changes preceding mTOR inhibition. Therefore, further studies are needed to understand whether there is an additional therapeutic target to further improve neuronal connectivity.

## Author contributions

AY designed, performed, supervised all experiments, and wrote the manuscript. RC and FCdA performed experiments on migration and contributed to writing the manuscript. TM performed Sholl analysis.

### Conflict of interest statement

The authors declare that the research was conducted in the absence of any commercial or financial relationships that could be construed as a potential conflict of interest.
